# Droplet Microfluidics‐Assisted Fabrication of Magnetite Nanoparticle Hybrid Microgels for Facile Protein Immobilization

**DOI:** 10.1002/cbic.202500958

**Published:** 2026-03-31

**Authors:** Talika A. Neuendorf, Anika Kaufmann, Russell J. Wilson, Kerstin G. Blank, Julian Thiele

**Affiliations:** ^1^ Institute of Physical Chemistry and Polymer Physics Leibniz Institute of Polymer Research Dresden Dresden Germany; ^2^ Department of Biomolecular and Self-Organizing Matter Institute of Experimental Physics Johannes Kepler University Linz Linz Austria; ^3^ Institute of Chemistry Otto von Guericke University Magdeburg Magdeburg Germany

**Keywords:** droplet microfluidics, enzyme immobilization, hybrid microgels, magnetite nanoparticles, magnetite-binding proteins

## Abstract

We introduce droplet microfluidics‐fabricated hybrid microgels composed of biotinylated acrylamide that is crosslinked with *N*,*N*’‐methylenebis(acrylamide). These microgels are further functionalized with streptavidin and loaded with magnetite nanoparticles (MNPs). During microfluidic processing, MNPs remain dispersed in an aqueous methylcellulose solution for over 4 h due to increased viscous drag, enabling stable droplet formation. In contrast, the MNPs sediment within a few minutes in pure aqueous solution. The resulting multifunctional hybrid microgels facilitate straightforward protein immobilization under mild conditions, for example, utilizing enzymes conjugated with streptavidin or recombinant fusion proteins of magnetite‐binding proteins. Simultaneously, our microgels enable the recovery of immobilized proteins through magnetic separation of the microgels from solution. The quantity of immobilized proteins can be regulated independently by varying the amount of coupled biotin or encapsulated MNPs. Using microgels containing different quantities of coupled biotin, we demonstrate binding of a streptavidin‐conjugated fluorescent dye and horseradish peroxidase. To confirm the availability of MNPs for magnetite‐binding proteins, a fusion protein of the magnetite‐binding protein Mad10 and super‐folder green fluorescent protein (sfGFP) was immobilized, and its fluorescence was detected.

## Graphical abstract

We present a versatile approach to uniform hybrid microgels containing magnetite nanoparticles (MNPs). Methylcellulose stabilizes MNPs in pre‐hydrogel suspensions, enabling microfluidic processing. Functionalization with biotin yields an experimental platform for enzyme immobilization, shown by immobilizing streptavidin‐horseradish peroxidase (HRP) and a colorimetric volume activity analysis using a 3,3',5,5'‐tetramethylbenzidine (TMB) oxidation assay. The microgels' magnetic properties enable simple recovery of bound enzymes.

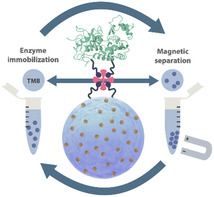

## 1 Introduction

Enzyme immobilization is a key application of magnetic nanoparticles (MNPs) in life sciences, as they can stabilize enzymes for repeated use and may even provide higher activity compared to applying enzymes in conventional buffer solution [1, 2, 3, 4, 5]. While their large surface‐to‐volume ratio is beneficial for immobilization purposes, MNPs are prone to aggregation to minimize their high surface energy by, for example, van der Waals interactions and electrostatic or magnetic attraction [6]. Furthermore, bare MNPs exhibit high chemical activity and are easily oxidized in air, typically resulting in a loss of magnetism and ability to form stable dispersions [7]. Therefore, stabilization strategies are essential for handling MNPs in aqueous solution and provide reliable enzyme immobilization.

A promising class of materials addressing this challenge are hybrid microgels, which contain inorganic or organic nanoparticles within their polymer network [8, 9]. Hybrid microgels are ideally suited for enzyme immobilization, as they can be readily functionalized to include specific binding sites for both non‐covalent and covalent attachment of enzymes. Iron oxide, especially magnetite (Fe_3_O_4_), is one of the most commonly used materials for MNPs for enzyme immobilization within microgels due to their ease of synthesis, low cost, and biocompatibility [5]. It was shown that bare MNPs in aqueous solution settled within a few minutes, while MNPs embedded inside hybrid microgels remained significantly more stable, with the first precipitation and agglomeration only observed after 24 h [10]. Studies have shown that these microgels can be conveniently separated from reaction media by applying an external magnetic field [9], thereby enhancing the microgels’ reusability and eliminating the need for energy‐ and time‐intensive centrifugation procedures [11], which may damage soft microgels through the applied mechanical stress.

However, the homogeneous encapsulation of nanoparticles inside a hydrogel matrix can be challenging as they tend to precipitate by gravitational sedimentation, leaving the area of research, despite its potential, fairly unexplored [9, 12]. The few methods describing the incorporation of MNPs inside microgels can generally be divided into two general approaches: (1) incorporation within pre‐fabricated microgels and (2) encapsulation during microgel fabrication. In the former case, incorporation has been mainly achieved through the direct deposition of MNPs in polymer microgels [10], in situ precipitation and stepwise growth of MNPs within the polymer network [13], as well as through solvent exchange [14]. Detrimental factors for broad applicability include labor‐intensive follow‐up steps required after microgel production, the limitation of MNP size due to the constraining polymer network, and the lack of control over homogeneous MNP distribution within the microgel. Alternatively, nanoparticles can be introduced during microgel formation, for example, by embedding them into the polymer network during emulsion polymerization [15]. However, without meticulous adjustment of the process parameters, bulk polymerization provides little control over nanoparticle loading, distribution, and microgel uniformity. A more promising method to ensure control over the microgels’ uniformity, porosity, and mechanical properties is droplet microfluidics. Poles et al. addressed the challenge of sedimentation by developing a shaking device that continuously homogenizes a suspension of MNPs within the precursor‐containing reservoir [12]. Although no sedimentation of the MNPs was observed during droplet formation, motor vibration caused an inhomogeneous droplet size distribution [12]. Another option is the use of ferrofluids as an additional dispersed phase for droplet and microgel formation [16], which, however, are limited in their functional flexibility and are often unstable in biological buffers due to their surface coating.

Here, we report the successful microfluidic fabrication of magnetic, hybrid microgels. These microgels are made from poly(acrylamide) (pAAm) with a tunable degree of biotin functionalization (biotinylation) and contain homogeneously distributed Fe_3_O_4_ nanoparticles. We first present a nanoparticle stabilization method for droplet microfluidics based on viscous drag. With this approach, the MNPs are uniformly dispersed within the aqueous AAm precursor solution, effectively preventing MNP sedimentation during experiments for at least over 4 h and eliminating the necessity for complex microfluidic flow cell design or supplementary equipment. In the next step, streptavidin‐conjugated horseradish peroxidase (HRP) is immobilized onto the biotinylated hybrid microgels, and volume activity (VA) is evaluated in dependence of the degree of biotinylation using a colorimetric assay. Furthermore, it is demonstrated that, in addition to gentle and efficient magnetic separation, the MNPs can be employed for selective immobilization of magnetite‐binding proteins, using the example of a recombinant fusion protein of sfGFP and an engineered variant of the protein Mad10 from *Desulfamplus magnetovallimortis* BW‐1.

## 2 Results and Discussion

### 2.1 Suspension Stability of Magnetite Nanoparticles in Aqueous Solution

**FIGURE 1:**
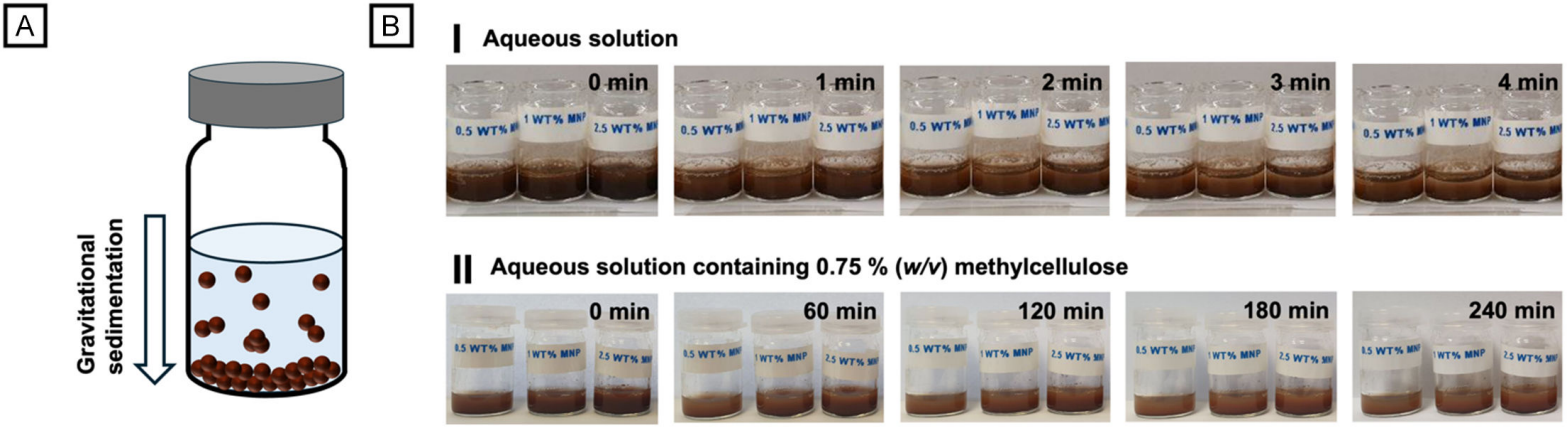
Sedimentation of MNPs over time in aqueous suspensions. (A) Scheme of gravitational sedimentation in aqueous suspension driven by the MNPs’ high density. (B) Comparative screening of sedimentation over time with varying concentrations of MNPs ranging from 0.5% to 2.5% (*w/w*) after 10 min of ultrasonication treatment in I) DI water and II) DI water supplemented with 0.75% (*w/v*) methylcellulose.

Despite their notable potential, MNPs are infrequently employed as additives in microfluidic experiments. This is primarily caused by agglomeration and gravitational sedimentation in aqueous solution, often caused by the inherently high density of MNPs. Consequently, during droplet microfluidics, the experimenter may face clogging of syringe needles and microchannels, disrupting droplet formation and causing an uneven distribution of MNPs within the produced microdroplets and microgels. This variability may cause the MNP content to fluctuate between excessive amounts and none at all, making the emerging hybrid microgels unsuitable for subsequent effective magnetic separation or any further application like enzyme immobilization (cf. Figure S1). To address these issues and achieve a stable, uniform dispersion of small aggregates, optimal preconditioning of the precursor solution is essential. To achieve this, we first perform simple bulk experiments in glass vials before sizing down to the desired microfluidic experiments. As an established, general method for nanoparticle deagglomeration into uniform dispersions, we employ acoustic cavitation via ultrasonication [17], and subject aqueous suspensions with varying MNP concentrations from 0.5% (*w/w*) up to 2.5% (*w/w*) to ultrasonic treatment for 10 min. As shown in Figure 1B‐I, initially, a visually homogeneous suspension is obtained. Nonetheless, the ultrasonic method offers only a temporary solution, as the substantial density difference between MNPs and deionized (DI) water (ρ_DI_ _water_ = 1 g cm^−3^; ρ_MNP_ ≈ 5 g cm^−3^) results in the re‐sedimentation and re‐agglomeration of the MNPs within minutes. Considering that a typical microfluidic experiment requires several hours to accumulate sufficient samples for further experimentation, the additional stabilization of MNPs in aqueous solution prior and during droplet/microgel formation is critical. Drawing inspiration from Real‐Time Deformability Cytometry (RT‐DC), a high‐throughput microfluidics method for continuously monitoring cell mechanics, we add methylcellulose to aqueous solutions of microgel precursors and MNPs [18]. As described by Stokes’ law, nanoparticle sedimentation velocity is inversely proportional to fluid viscosity. Although increasing the viscous drag is beneficial for dispersion stability, an excessive viscosity contrast between the fluid streams of continuous and dispersed phases must be prevented to maintain laminar flow within the microfluidic flow cell. Therefore, a 0.75% (*w/v*) methylcellulose solution is used for all experiments conducted in this study. By adding methylcellulose, the MNP stability in suspension is greatly improved from a few minutes to over 4 h for MNP concentrations ranging from 0.5 up to 2.5% (w/w) (Figure 1B‐II).

### 2.2 Droplet Microfluidics‐Assisted Fabrication of Biotinylated Polymer Hybrid Microgels

**FIGURE 2:**
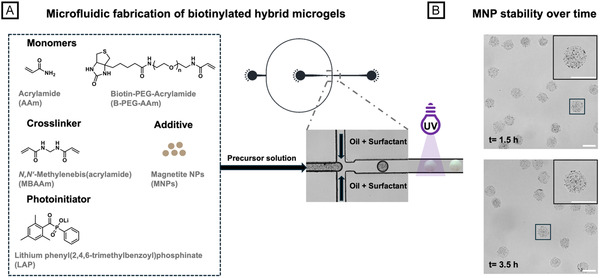
Fabrication and stability of biotin‐functionalized hybrid microgels loaded with MNPs. (A) Scheme illustrating droplet microfluidics‐assisted fabrication of biotin‐functionalized, poly(acrylamide)‐based hybrid microgels with MNPs. HµGel‐XB denotes biotinylated microgel, where X indicates biotin‐PEG‐acrylamide concentration in the precursor solution in % (*w/w*). Following ultrasonic treatment, the precursor solution is injected into microfluidic channels with flow‐focusing design (as shown in the AutoCAD design). Crosslinking of water‐in‐oil (W/O) emulsion droplets occurs via UV irradiation within the outflow tubing. (B) MNP stability in the precursor solution over time demonstrated by bright‐field microscopy images of purified microgels containing 1% (*w/w*) MNP and 2.5% biotin (HµGel‐2.5B) after 1.5  and 3.5 h of experimentation, respectively. Insets show uniform distribution of MNPs within the microgels and reveal consistent MNP loading during microfluidic processing over time. All scale bars denote 150 μm.

Biotinylation offers a facile method for enhancing the versatility of MNP hybrid microgels enabling their application in enzyme immobilization. Reproducibility and, thus, highly uniform microgel size are crucial factors as each microgel can be regarded as a self‐contained reaction system. Therefore, in this study, droplet microfluidics is employed for the fabrication of microgels. For introducing biotin groups into the hybrid microgel network, the monomers acrylamide (AAm, monomer 1) and biotin‐PEG‐acrylamide (B‐PEG‐AAm, monomer 2) are co‐polymerized via UV irradiation with *N*, *N’*‐methylenebis(acrylamide) (MBAAm) serving as a crosslinker (Figure 2A). A total of 10% (*w/w*) monomer is added to all precursor solutions, with the concentration of B‐PEG‐AAm varying from 1.25% to 5% (*w/w*) to examine the impact of biotin concentration on enzyme immobilization efficiency. The upper concentration limit of B‐PEG‐AAm in these precursor formulations is 5% (*w/w*). Beyond this concentration, we do not observe any UV‐induced gelation of the precursor microdroplets, likely due to steric hindrance caused by the biotinylated monomer. While our preliminary studies on sedimentation of MNPs reveal that contents up to 2.5% (*w/w*) can be stabilized in aqueous methylcellulose solution, we limit the MNP concentration to 1% (*w/w*) across all fabricated microgel populations. This decision is made to ensure proper passage of UV light during irradiation while maintaining effective magnetic separation of the hybrid microgels. To prevent MNP leakage from microgel precursor droplets during microfluidic processing, UV irradiation is performed shortly after W/O emulsion droplet formation in the outflow tubing using a 3D‐printed, custom‐built UV irradiation device that ensures even illumination at a fixed distance of 2 cm towards the tubing [19]. Using a flow‐focusing device with a microchannel cross‐section of 100 µm at the droplet‐forming nozzle, we obtain emulsion droplets 108 ± 3 µm in diameter for hybrid microgels with 1.25% (w/w) B‐PEG‐AAm (HµGel‐1.25B), 109 ± 3 µm in diameter for hybrid microgels with 2.5% (*w/w*) B‐PEG‐AAm (HµGel‐2.5B), and 111 ± 5 µm in diameter for hybrid microgels with 5% (w/w) B‐PEG‐AAm (HµGel‐5B) (Table 1).

**TABLE 1:** Resulting emulsion droplet and microgel size in dependence on monomer ratio and time of experimentation.

Microgel population	**Monomer 1 : Monomer 2 ratio** a	**Emulsion droplet diameter** b, μ**m**	**Microgel diameter** c, μ**m**	**Swelling degree** d **, %**
HμGel‐1.25B	7 : 1	108 ± 2	129 ± 6	19.5 ± 5.3
HμGel‐2.5B	3 : 1	109 ± 2	136 ± 8	25.6 ± 8.9
HμGel‐5B	1 : 1	112 ± 7	122 ± 5	22.2 ± 10.1

a Monomer 1 is AAm, monomer 2 is B‐PEG‐AAm.

b n_droplet_ = 45.

c n_microgel_ = 45, collected after 1.5 h into the experiment and analyzed after 24 h swelling in water.

d *n* = 45.

After purification through emulsion breakage via addition of isopropanol, subsequent transfer to water, and swelling in water for 24 h, microgel diameter and swelling degree were analyzed (cf. Table 1). As the fraction of B‐PEG‐AAm in the microgel network increases, an initial rise in swelling degree from 19.5 ± 5.3% for HµGel‐1.25B to 25.6 ± 8.9% for HµGel‐2.5B is observed (cf. Table 1). We believe that introducing biotin as a rather bulky side chain disrupts the microgel network and reduces effective crosslink density. The resulting increase in free volume and hydrogel mesh size favors water uptake, leading to greater swelling with increased biotin concentration [20]. However, a slight decrease in swelling to 22.2 ± 10.1% is observed for HµGel‐5B. This non‐monotonic swelling behavior likely arises from competitive molecular interactions within the microgel network. We suggest that as biotin concentration increases, its hydrophobic nature and added steric constraints take precedence, restricting the microgel’s expansion during swelling. Bright‐field microscopy image analysis reveals that no free MNPs are present in the suspension after extended swelling in water. Furthermore, as exemplarily shown for HµGel‐2.5B, a time‐independent uniform distribution of MNPs as well as consistent MNP loading is observed in microgels collected after 1.5 hr and after 3.5 hr. This confirms the successful stabilization of the MNPs in the precursor solution and the homogeneous encapsulation of the MNPs in the microgel network (cf. Figure 2B).

### 2.3 Biotin Availability in Hybrid Microgels

**FIGURE 3:**
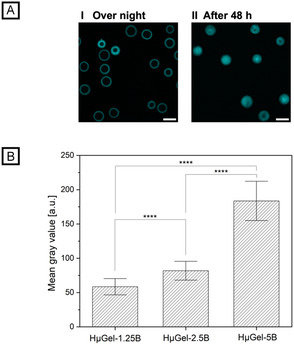
Fluorescent labeling of biotinylated hybrid microgels with streptavidin‐Atto 425 conjugate. (A) CLSM images of streptavidin‐Atto425 after incubation with HµGel‐2.5B overnight and after 48 h. The scale bars denote 150 µm. (B) Calculated mean gray values of streptavidin‐Atto425 on hybrid microgels with varying biotin content (*n* = 30 ± s.d.) after overnight incubation. Two‐sample *t* test, where ****p < 0.0001.

Efficient enzyme immobilization requires that enzymes can diffuse through the microgel and that biotin moieties are available both on the surface and throughout the microgel network. Biotin availability is confirmed using streptavidin binding, being among the strongest non‐covalent interactions and highly specific [21]. For this purpose, the hybrid microgel species HµGel‐1.25B, HµGel‐2.5B, and HµGel‐5B are incubated overnight with streptavidin‐conjugated Atto 425 dye, followed by thorough washing to remove unbound streptavidin. Confocal laser scanning microscopy (CLSM) images of HµGel‐2.5B in Figure 3A‐I show that overnight incubation primarily leads to saturation with streptavidin‐Atto 425 on the microgel surface and the outer layers. Because streptavidin binds strongly to biotin (Kd ≈ 10^−14^) [22], it initially attaches to the microgel exterior where biotin groups are readily available. Its size (molecular mass ≈ 60 kDa) and steric hindrance slow down the diffusion of further streptavidin into the network. However, after 48 h, a uniform distribution of the fluorescent dye is observable within the hydrogel (Figure 3A‐II). We suggest the formation of a diffusion‐driving concentration gradient, with a high concentration of streptavidin and few free biotin groups on the microgel exterior, and a low concentration of streptavidin with many available biotin groups within the microgel. The system is in dynamic equilibrium, where streptavidin may slowly dissociate and penetrate further into the microgel volume until a homogeneous distribution is reached. We also investigated how varying biotin concentrations affect streptavidin binding within the hybrid microgels. To do this, the average gray values (fluorescence intensity values) across the cross‐section of CLSM images are determined. For the lowest biotin concentration in HµGel‐1.25B, a mean gray value of 58.5 ± 11.9 is recorded, which increases with rising biotin concentrations to 81.8 ± 13.7 for HµGel‐2.5B and ultimately to 183.6 ± 28.6 for HµGel‐5B (Figure 3B). Thus, the biotin is not only homogeneously distributed throughout the network, but adjusting the precursor solution also enables direct control over the concentration of bound streptavidin–Atto 425 within the microgel network.

### 2.4 Immobilization of Streptavidin‐Conjugated HRP and Colorimetric Activity Assay

**FIGURE 4:**
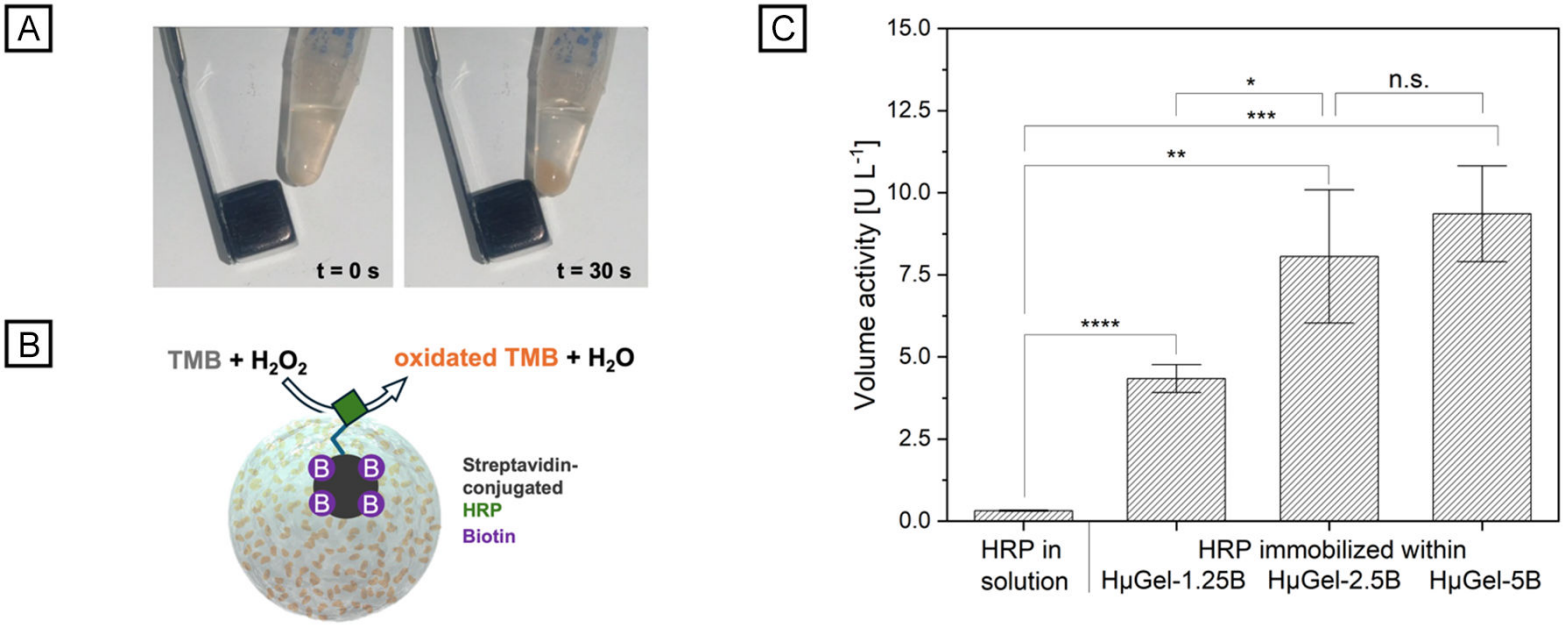
Magnetic separation and enzymatic activity of biotinylated hybrid microgels containing immobilized HRP. (A) Magnetic separation of the hybrid microgels within 30 s. The supernatant can easily be collected by pipetting. (B) Scheme of immobilizing streptavidin‐conjugated HRP on hybrid microgels and the respective activity assay based on TMB oxidation in the presence of H_2_O_2_. (C) Volume activity of free HRP and immobilized HRP in hybrid microgels with varying biotin content (*n* = 3 ± s.d.). Two‐sample t test, where n.s.; not significant (*p* > 0.5), *p < 0.1, **p < 0.01, ***p < 0.001, ****p < 0.0001.

To demonstrate the usability of the hybrid microgels for enzyme immobilization, they were incubated overnight with streptavidin‐conjugated HRP at 4°C. One major advantage of our hybrid microgels over conventional microgels is the ability to purify them via mild magnetic separation. In a single step, the hybrid microgels can be separated from the supernatant within 30 s (Figure 4A). Besides speed and simplicity, this method prevents microgel deformation and enzyme denaturation by significantly reducing shear forces as compared to conventional separation by centrifugation, enabling the enzyme‐loaded microgels to be reused for further experiments [23, 24]. Particularly in the case of complex and cost‐intensive enzymes, as well as in the context of sustainability efforts, the reuse of such enzymes is becoming increasingly important. After purification and removal of unbound enzyme, the enzyme activity is assessed using a colorimetric assay with 3,3′, 5,5′‐tetramethylbenzidine (TMB) as the chromogenic substrate. In the presence of hydrogen peroxide (H_2_O_2_), HRP catalyzes the oxidation of the colorless TMB into its orange‐colored diimine (Figure 4B). The formation of the diimine is spectrophotometrically monitored at 465 nm over 4 min. To evaluate the effect of the microgel on enzyme stability and activity in general, the enzyme activity in free solution is determined for comparison. For this purpose, the same amount of enzyme (20 ng), which was used for enzyme immobilization in microgels, was also kept in solution (PBS) under identical conditions overnight (thermoshaker, 4°C, 400 rpm). After incubation, the microgels were washed to remove unbound enzyme, resulting in a lower enzyme amount within the microgels compared to the free enzyme in solution. Quantitative calculation of enzyme concentration in microgels after immobilization at such low concentrations is challenging and susceptible to high errors. Therefore, the VA of the solution (cf. Equation S1) was determined, which is independent of the enzyme quantity and allows for a comparison of activity within the same volume. Generally, the VA of HRP is significantly higher after immobilization for all hybrid microgels, as for HRP in solution, where the VA is 0.32 ± 0.02 μL^−1^. The microgel functions as a protective and stabilizing reaction matrix [25]. Immobilization enhances enzyme activity by reducing conformational changes and preventing aggregation, thereby mitigating enzyme denaturation. Furthermore, substantially higher local substrate concentrations can be attained within the microgel in comparison to non‐immobilized enzyme in solution [26, 27]. VA values differ depending on the biotin content of the HµGel‐XB. Here, HµGel‐1.25B has the lowest VA, with 4.34 ± 0.42 μL^−1^, which is still 13.5‐fold higher than that of free HRP. The VA of HµGel‐2.5B is 8.06 ± 2.03 μL^−1^ and thus 25‐fold higher than for free HRP and significantly higher than for HµGel‐1.25B. With increasing biotin concentration from HµGel‐1.25B to HµGel‐2.5B, there is a significant increase in VA, as more binding partners are available for HRP immobilization, allowing for more substrate conversion. Although, as shown in Figure 3, the concentration of bound streptavidin‐Atto 425 increases with increasing biotin concentration in the microgel network, this effect cannot be observed to the same extent for streptavidin‐HRP. A further increase in biotin does not significantly change the amount of immobilized HRP, as indicated by a VA of 9.36 ± 1.46 μL^−1^ for HµGel‐5B, which is 29‐fold higher than for free HRP and, therefore, not significantly higher than for HµGel‐2.5B. Since the porous microgel network allows the diffusion of small substrate molecules, enzyme immobilization should not limit access to the substrate. For this reason, we hypothesize that the larger hydrodynamic radius of streptavidin‐HRP molecules may lead to their interference, resulting in reduced binding efficiency and enzyme activity per molecule. Additionally, with limited diffusion of streptavidin‐HRP into the microgel volume due to steric hindrance, streptavidin binding sites might already be saturated despite increased biotin concentration. Besides that, the enzyme saturation point could be reached, where a further increase in enzyme amount did not result in higher VA.

### 2.5 Immobilization of Magnetite‐Binding Proteins

**FIGURE 5:**
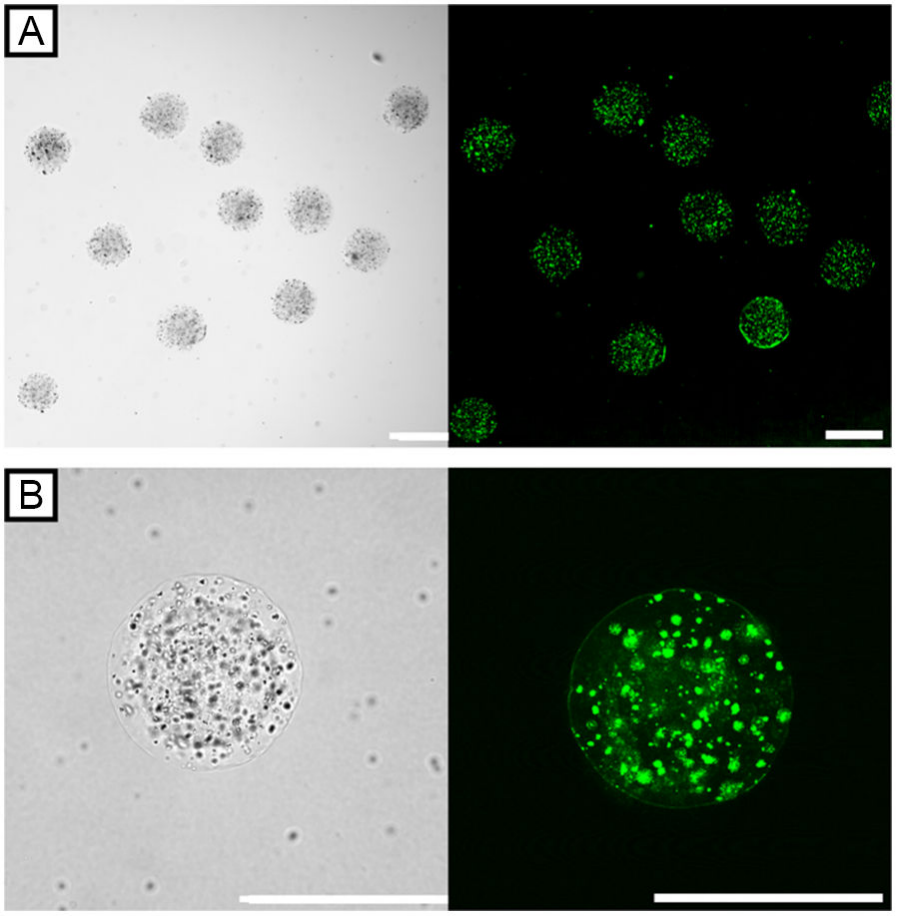
Immobilization of sfGFP‐Mad10trunc‐His fusion protein on MNPs embedded inside pAAm‐based hybrid microgels. (A) 10× and (B) 40× bright‐field and corresponding CLSM microscopy images showing the selective binding of sfGFP‐Mad10trunc‐His to MNPs within HµGel‐1.25B after 3 days of incubation. All scale bars denote 150 µm.

Functional modularity concerning the immobilized enzyme system typically requires a multi‐step functionalization effort and is particularly limited for large substrates due to diffusional barriers. Interestingly, MNPs do not only serve as an additive for magnetic separation of microgels, but – due to our well‐controlled processing and stabilization of MNPs inside the pAAm‐based hybrid microgels – can also be explored as a platform for functionalization with fusion proteins of magnetite‐binding proteins. In contrast to conventional covalent immobilization of enzymes on metal oxide carrier materials, functionalization of the protein with a magnetite‐specific affinity tag prevents uncontrolled, non‐specific binding of the protein, leaving its structure and function unaffected. Making use of the magnetite binding protein Mad10 from the magnetotactic bacterium *Desulfamplus magnetovallimortis* BW‐1, which binds to magnetite surfaces strongly and irreversibly [28], we demonstrate a simple, one‐step functionalization method of MNPs embedded in our microgels. As a proof of concept, HµGel‐1.25B was incubated with a fusion protein of Mad10 and superfolder GFP (sfGFP‐Mad10trunc‐His; 3 mg mL^−1^) at 4°C. After purification, we confirm successful and selective fluorescent labeling of the MNPs by CLSM (Figure 5A,B).

## 3 Conclusion

In this study, we have implemented a method to overcome the challenge of stabilizing MNPs in microfluidic experiments during W/O emulsion droplet and microgel formation. By introducing methylcellulose to aqueous precursor solutions, the sedimentation time of MNPs increases from a few minutes to several hours compared to DI water, due to the associated higher viscous drag. As a result, up to 2.5% (w/w) MNPs can be stabilized and continuously processed by droplet microfluidics. We see great potential in this simple method, as it ensures uniform MNP distribution and reproducible microgel quality in microfluidics, which is key for biological and sensor applications. Further, no specialized equipment is needed, and the method can be easily adapted to various types of nanoparticles.

Combining the approach with biotin functionalization, the hybrid microgels are suitable for enzyme immobilization using the biotin–streptavidin interaction, as successfully shown using streptavidin‐HRP as a model enzyme. Here, a significant increase in VA was demonstrated when moving from free enzymes in solution to microgel‐immobilized enzymes. The hybrid microgels can be readily separated from the supernatant using a simple magnet, eliminating the need for centrifugation, and thus preserving both enzyme activity and microgel structure.

A key advantage of our hybrid microgels lies in their dual orthogonal functionality. In addition to immobilization based on biotin‐streptavidin affinity, we demonstrate that the encapsulated MNPs enable site‐specific immobilization of magnetite‐binding proteins, using the example of Mad10. This duality of immobilization offers selective and tailored co‐immobilization of enzymes with high spatial control within a single microgel. Overall, we believe that our hybrid microgels are a powerful platform for simple, one‐step enzyme immobilization and separation, making it accessible for a broad range of applications in cell‐free biosynthesis and synthetic biology [29, 30, 31, 32].

## 4 Experimental Section

### 4.1 Materials

All chemicals were used without further purification unless stated otherwise. Acrylamide (Aam, ≥99%), *N*, *N’*‐methylenebisacrylamide (BIS‐AAm, ≥99.5%) and methylcellulose (viscosity: 1,500 cP) were purchased from Sigma–Aldrich (St. Louis, MO,USA). Biotin‐PEG‐acrylamide (B‐PEG‐AAm, *M*_W_ = 10,000; ≥95%) was purchased from Biopharma PEG Scientific Inc. (Watertown, MA, USA). Lithium phenyl‐2,4,6‐trimethylbenzoylphosphinate (LAP, >98%) was purchased from TCI Chemicals (Tokyo, Japan). Iron(II, III) oxide (Magnetite, 20–30 nm, ≥98%) and 3 M Novec 7500 (HFE 7500, >99%) were purchased from IoLiTec Ionic Liquids Technologies GmbH (Heilbronn, Germany). Streptavidin Atto 425 conjugate was purchased from ATTO‐TEC GmbH (Siegen, Germany). Streptavidin‐horseradish peroxidase conjugate (streptavidin‐HRP) was purchased from Jackson ImmunoResearch Laboratories Inc. (West Grove, PA, USA). Hydrogen peroxide (H_2_O_2_, 30%, stabilized) and 3,3^′^, 5,5^′^‐tetramethylbenzidine (TMB) were purchased from Carl Roth GmbH & Co. KG (Germany). Deionized water (DI water) with a resistance of 18.2 MΩ cm was prepared in a Milli‐Q Direct 8 water purification system (Merck, Millipore, Burlington, MA, USA). The phosphate‐buffered saline (PBS) buffer was composed of KH_2_PO_4_ (1.7 mM), Na_2_HPO_4_ · 2H_2_O (5 mM), and NaCl (154 mM) and adjusted to pH 7.4.

### 4.2 Time‐Dependent Gravitational Sedimentation of Magnetite Nanoparticles in Aqueous Solution

First, a methylcellulose solution (1%, w/v) was prepared, diluted to 0.75% (*w/v*) with DI water, and filtered through a Nylon syringe filter (Rotilabo‐syringe filter, Nylon, pore size 0.45 µm, Carl Roth GmbH & Co. KG, Karlsruhe, Germany). For studying time‐dependent gravitational sedimentation, MNPs (mean diameter 20–30 nm) were suspended at varying concentrations of 0.5%, 1%, and 2.5% (*w/w*) in 2 mL DI water or the methylcellulose solution. Prior to use, the MNP suspension‐containing glass vials were ultrasonicated for 10 min. Subsequently, images were taken every minute for DI water samples and every hour for methylcellulose samples to observe the sedimentation behavior of the MNPs.

### 4.3 Microfluidic Flow Cell Fabrication and Experimental Setup Design

Combined photo‐ and soft lithography was employed for manufacturing poly(dimethylsiloxane)‐based (PDMS) microfluidic flow cells. First, a master structure was created by spin‐coating the negative photoresist SU‐8 2050 (Micro Resist Technology, Berlin, Germany) onto the polished side of a 3‐inch silicon wafer (Siegert Wafer, Aachen, Germany). Using a mask aligner (MJB3, Süss MicroTec, Garching, Germany), the desired structure was illuminated onto the resist through a printed photomask. Non‐illuminated and, therefore, non‐hardened photoresist was removed with a developer (mrDev 600, Micro Resist Technology, Berlin, Germany) after post‐exposure and hard‐bake steps. The target microchannel height and width at the droplet‐forming nozzle were 100 µm. Subsequently, the master structure was replicated in PDMS. After combining the PDMS base and crosslinker mixture at a 10:1 (*w/w*) ratio, they were mixed and degassed in a planetary centrifugal mixer (ARE‐250, Thinky, Laguna Hills, CA, USA) and finally poured onto the master structure and cured at 80°C for 1 h. After curing, the PDMS replica was cut out and peeled off. Tubing access ports for in‐ and outflow of continuous and dispersed phases were punched into the PDMS replica utilizing a biopsy punch (diameter: 1.0 mm, KAI Medical, Solingen, Germany). After thorough cleaning with isopropanol, the microchannel‐bearing side of the PDMS replica and a microscopy glass slide (76 × 26 mm) were bonded to each other after oxygen plasma treatment (100 W, 20 s, MiniFlecto 10, Plasma Technology GmbH, Herrenberg, Germany).

Prior to all microfluidics experiments, the microchannel surface was hydrophobized by flushing with a 1% (*v/v*) solution of (tridecafluoro‐1,1,2,2‐tetrahydrooctyl)trichlorosilane (Gelest, Morrisville, PA, USA) in Novec 7500 (IoLiTec, Heilbronn, Germany). For microfluidic experiments, the flow cells were connected to high‐precision syringe pumps (Harvard Apparatus Pump 11 p Plus Elite, Harvard Apparatus, Holliston, MA, USA) via PE tubing (inner diameter: 0.38 mm, outer diameter: 1.09 mm, Instech Laboratories Inc., Leipzig, Germany). A 1,000 µL gastight glass syringe (1750 TLL SYR, Hamilton, Franklin, IN, USA) was used for the dispersed phase (DP), and a 10 mL disposable syringe (BD Luer‐Lok tip, Becton Dickinson, Franklin Lakes, NJ, USA) was used for the continuous phase (CP). Droplet formation was monitored through a high‐speed camera (Miro C110, Vision Research Inc., Wayne, NJ, USA) coupled to an inverted bright‐field microscope (10× objective lens, air, Axio Vert.A1, Carl Zeiss, Oberkochen, Germany).

### 4.4 General Procedure of Biotin‐Functionalized Acrylamide‐Based Microgel Fabrication

For fabricating biotin‐functionalized hybrid microgels (B‐HµG), a 1% (*w/v*) methylcellulose solution was prepared and filtered through a Nylon syringe filter (Rotilabo‐syringe filter, Nylon, pore size 0.45 µm, Carl Roth GmbH+Co. KG, Karlsruhe, Germany) before usage. For the dispersed phase, two separate solutions were prepared. 6 mg of MNP powder was dispersed in 132.5 µL deionized water by 10 min of ultrasonication. Meanwhile, by centrifugation, AAm (Monomer 1) and B‐PEG‐AAm (Monomer 2) were dissolved in 397.5 µL of a 1% (*w/v*) methylcellulose solution. The accumulated amount of both monomers was maintained at 60 mg (equivalent to 10% *w/w*) for all experiments with varying ratios (Table 2).

**TABLE 2:** Overview of monomer amounts used for the fabrication of biotinylated hybrid microgels.

Sample^a^	Monomer 1, mg	Monomer 2, mg
HμGel‐1.25B	52.5	7.5
HμGel‐2.5B	45	15
HμGel‐5B	30	30

a HμGel‐XB denotes “biotinylated hybrid microgel”; X indicates the amount of B‐PEG‐AAm in % (*w/w*) in the overall formulation.

Then, 2.5 mg of bis‐AAm (crosslinker) and 1.5 mg of LAP (photoinitiator) were dissolved as well in the 1% (w/v) methylcellulose solution by centrifugation. Finally, both solutions (DI water and 1% (*w/v*) methylcellulose solution) were combined, yielding a final concentration of 0.75% (*w/v*) methylcellulose in the aqueous solution. Due to its high viscosity, the DP was injected at a flow rate of 800 µL h^−1^ and emulsified at the junction into droplets by the CP at a flow rate of 50 µL h^−1^. The CP consisted of a triblock copolymer surfactant (PFPE‐PEG‐PFPE, RAN Biotechnologies, Beverly, MA, USA, 2% w/w) dissolved in HFE 7500. Droplet crosslinking was performed during fabrication within the outflow tubing by UV irradiation at 20% relative UV intensity of an OmniCure S1500 (200 W, 250–450 nm, 44.6 mW cm^−2^, Asslar, Germany) connected to the tubing by a light guide through a custom‐built, 3D‐printed fixation device to ensure even illumination at a fixed distance of 2 cm. The microgel‐containing emulsions were collected in parafilm‐sealed 1.5 mL Eppendorf tubes at 25‐min intervals. For purification, the surfactant‐laden, fluorinated oil phase was removed, and 200 μL of isopropanol was added. The washing step was repeated twice more. Subsequently, the microgels were transferred into water by adding 200 µL of deionized water. The washing step was repeated three more times. Bright‐field microscopy images were recorded on a TCS SP8 microscope (Leica, Wetzlar, Germany). At least 45 droplets and microgels were evaluated manually using the software Fiji for emulsion droplet and microgel size analysis [33]. The mean diameter and swelling degree, along with their respective standard deviations, were calculated based on the obtained data using Origin. Two‐sample t‐tests were performed in Origin.

### 4.5 Fluorescent Labeling of Biotinylated Hybrid Microgels

HμGel‐1.25B, HμGel‐2.5B, and HμGel‐5B microgels were transferred into 1× PBS buffer and concentrated by centrifugation. 25 µL of each concentrated microgel suspension was incubated overnight with 25 µL of Streptavidin Atto 425 conjugate (1 mg mL^‐ 1^ in DI water) in a thermoshaker (4°C, 400 rpm). To remove unbound fluorescently labeled streptavidin, the microgels were washed with 100 µL of 1x PBS, concentrated by centrifugation, and the supernatant was discarded. This washing step was repeated two more times. CLSM images were recorded on a TCS SP8 microscope (Leica, Wetzlar, Germany). By applying line scans, cross‐sections of 30 microgels for each of the three microgel batches were evaluated manually using Fiji for fluorescence characterization [33]. Fluorescence intensity was calculated using two gray value maxima along the microgel’s cross‐section [30].

### 4.6 Immobilization and Activity Assay of HRP

To analyze the amount of free enzyme in solution, 20 ng of streptavidin‐HRP conjugate were incubated overnight in 200 µL of 1x PBS in a thermoshaker (4°C, 400 rpm). For enzyme immobilization, HμGel‐1.25B, HμGel‐2.5B, and HμGel‐5B microgels were transferred into 1x PBS buffer and concentrated by centrifugation. 20 µL of each concentrated microgel suspension was incubated overnight with 20 ng of streptavidin‐HRP in 200 µL of 1× PBS in a thermoshaker (4°C, 400 rpm). To remove non‐immobilized streptavidin‐HRP, the microgels were washed by adding 200 µL of 1× PBS, concentrated by magnetic separation utilizing a neodymium magnet in close proximity to the hybrid microgel‐containing Eppendorf tube (cf. Figure 4A), and 200 µL of the supernatant was discarded. That way, the volume was kept constant at 200 µL during the washing steps. After repeating the washing step for two more times, a colorimetric analysis of HRP VA was performed via a TMB oxidation assay. Stock solutions of 1 mM H_2_O_2_ and 1 mM TMB were prepared immediately before use. For each assay, 100 µL of reaction mixture was prepared, comprising 70 µL of 1× PBS, 5 µL of TMB (1 mM, in DMSO), 5 µL of H_2_O_2_ (1 mM), and either 20 µL of overnight‐incubated streptavidin‐HRP or 20 µL of streptavidin HRP‐immobilized HµGel‐1.25B, HµGel‐2.5B, or HµGel‐5B, each incubated overnight. Each assay was conducted immediately after mixing in triplicate using a Tecan Infinite M200 PRO plate reader (96‐well plate, Tecan Trading AG, Switzerland), measuring absorbance at 465 nm for a duration of 4 min. To adjust the absorbance values, two blank measurements were recorded with either 20 µL of 1× PBS or 20 µL of non‐immobilized HµGel‐1.25B, HµGel‐2.5B, or HµGel‐5B, which were subtracted from the raw data.

### 4.7 Immobilization of sfGFP‐Mad10trunc‐His Fusion Protein

Information on gene design, recombinant protein expression, and protein purification can be found in the SI. Prior to incubation, a solution of sfGFP‐Mad10trunc‐His in phosphate–based interaction buffer (pH = 8; see Supporting Information) was concentrated by ultrafiltration using a centrifugal filter (AmiconUltra‐0.5 mL, Ultracel ‐ 10K, Merck Millipore Ltd., Ireland) to a concentration of 3.6 mg mL^−1^. Exemplarily, for protein immobilization, HµGel‐1.25B microgels were transferred into interaction buffer and concentrated by centrifugation. 6 µL of the concentrated microgel suspension was incubated overnight with 30 µL sfGFP‐Mad10trunc‐His (final protein concentration: 3 mg mL^−1^) in a thermoshaker (4°C, 400 rpm). After 3 days of storage at 4°C, non‐immobilized protein was removed by washing the microgels twice with 100 µL of interaction buffer. After concentration by centrifugation, the supernatant was discarded, and enzyme‐functionalized microgels were imaged by CLSM.

## Supporting Information

Additional supporting information can be found online in the Supporting Information section. Additional references are provided in the Supporting Information [34, 35]. **Supporting Figure S1**: Bright‐field microscopy images of purified hybrid microgels containing magnetite‐nanoparticles (MNPs) obtained from a microfluidic experiment with an acrylamide‐based precursor solution serving as the dispersed phase. The NMPs are used without pretreatment or stabilization. Gravity‐induced sedimentation and flow interruption due to the MNPs being of a different density than their surrounding media result in their uneven distribution inside the microgel volume. Insets show examples of microgels of one collected batch with varying MNP loading ranging from no loading at all (I) to pronounced loading (III). All scale bars denote 100 μm. **Supporting Figure S2**: Reducing SDS‐PAGE (15%) showing expression and purification of sfGFP‐Mad10trunc‐His with L: lysate, I: insoluble protein, S: soluble protein, F: flow‐through, W: washing buffer, and E: eluted protein. The black arrow indicates the sfGFP‐Mad10trunc‐His band on the gel.

## Conflicts of Interest

The authors declare no conflicts of interest.

## Supporting information

Supplementary Material

## Data Availability

The data that support the findings of this study are available from the corresponding author upon reasonable request.
