# Supplementary material for: Droplet Microfluidics‐Assisted Fabrication of Magnetite Nanoparticle Hybrid Microgels for Facile Protein Immobilization
Source: Chembiochem. 2026 Mar 31;27(7):e202500958. doi: 10.1002/cbic.202500958 (PMC13039770; doi:10.1002/cbic.202500958)
Supplement: Supplementary file 1 — Supplementary Material [file CBIC-27-e202500958-s001.pdf]

Supporting Information

Droplet Microfluidics-assisted Fabrication of Magnetite-Nanoparticle Hybrid Microgels for Facile Protein Immobilization

Talika A. Neuendorf,[b] Anika Kaufmann,[b] Russell J. Wilson,[c] Kerstin G. Blank,[c] Julian Thiele*[a,b]

[a] Institute of Chemistry, Otto von Guericke University Magdeburg, 39106 Magdeburg, Germany

[b] Institute of Physical Chemistry and Polymer Physics, Leibniz Institute of Polymer Research Dresden, 01069 Dresden, Germany

[c] Department of Biomolecular and Selforganizing Matter, Institute of Experimental Physics, Johannes Kepler University Linz, 4040 Linz, Austria

* E-mail: julian.thiele@ovgu.de

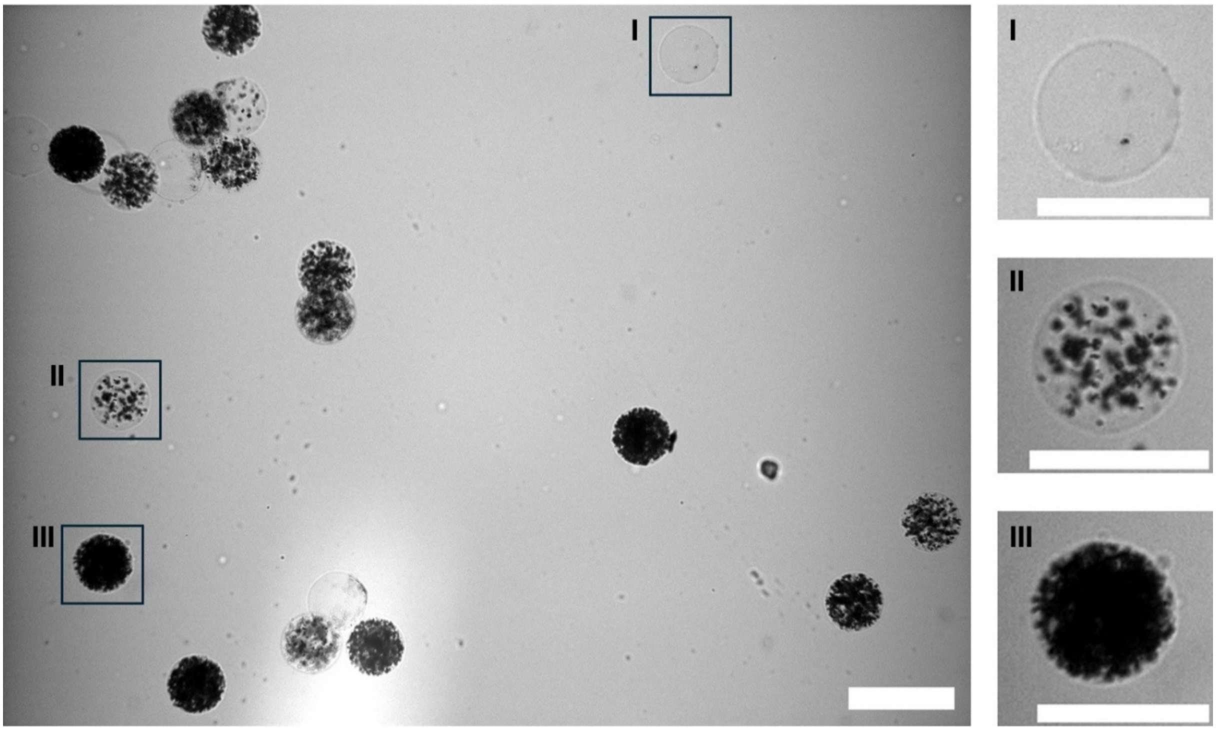

Figure S1: Bright-field microscopy images of purified hybrid microgels containing magnetite-nanoparticles (MNPs) obtained from a microfluidic experiment with an acrylamide-based precursor solution serving as the dispersed phase. The NMPs are used without pretreatment or stabilization. Gravity-induced sedimentation and flow interruption due to the MNPs being of a different density than their surrounding media result in their uneven distribution inside the microgel volume. Insets show examples of microgels of one collected batch with varying MNP loading ranging from no loading at all (I) to pronounced loading (III). All scale bars denote 100 µm.

Calculation of the Volume Activity of HRP in the Colorimetric Activity Assay

Equation S1: Calculation of the volume activity (VA) of Streptavidin-conjugated HRP in the colorimetric activity assay with 3,3',5,5'-tetramethylbenzidine as the chromogenic substrate, where $\frac{\Delta Abs}{\Delta t}$ = rate of change in absorbance over time, V_{total} = total assay volume, V_{Enzyme} = total enzyme volume in assay, ε_{465} = extinction coefficient at 465 nm, d_{well} = plate well thickness, DF = dilution factor.

$$VA = \frac{\frac{\Delta Abs}{\Delta t} \cdot V_{total}}{V_{Enzyme} \cdot \varepsilon_{465} \cdot d_{well} \cdot DF} \tag{1}$$

Gene Design of sfGFP-Mad10trunc-His

The expression of the fusion protein sfGFP-Mad10trunc-His was performed as previously described.[1] The gene designed for the expression of sfGFP-Mad10trunc-His encodes a 40.3 kDa recombinant protein. From *N*- to *C*-terminus, the gene contains a superfolder green fluorescent protein (sfGFP) using amino acids **1-229**[1,2], a linker composed of an enterokinase cleavage site (DDDDK), the magnetite-binding protein Mad10 truncated to amino acids **2-108** (Mad10trunc)**,** and a 6x His-tag. The gene was obtained through gene synthesis and has been codon-optimized for expression in *E. coli*. The gene was flanked by NcoI and EagI restriction sites and cloned into a pET-28a(+) vector (Novagen, Merck Millipore). The amino acid and DNA sequences are given below. In the DNA sequence, the restriction sites for NcoI and EagI are highlighted in underlined text.

Amino Acid Sequence

MGSKGEELFTGVVPILVELDGDVNGHKFSVRGEGEGDATNGKLTLKFICTTGKLPVPWPTLVTTLTYGVQCFSRYPDHMK
QHDFFKSAMPEGYVQERTISFKDDGTYKTRAEVKFEGDTLVNRIELKGIDFKEDGNILGHKLEYNFNSHNVYITADKQKNG
IKANFKIRHNVEDGSVQLADHYQQNTPIGDGPVLLPDNHYLSTQSVLSKDPNEKRDHMVLLEFVTAAGIGSDDDDKEFGR
GIRYTMYNLGDIFVDKLESMWNSTKISTKGIRLTHNIRKLRSIKEEQERKLSGRVLELRESYPELEIFKDDELSKLFSEIDAIN
RELDSYIEERDEILYPTGRSVASHHHHHH

DNA Sequence

CCATGGGCAGCAAGGGTGAGGAACTATTCACGGGCGTAGTTCCAATTCTCGTAGAGCTGGACGGGGATGTCAACGG
CCACAAATTTTCTGTGCGCGGGGAGGGGGAGGGGGATGCTACCAACGGCAAATTGACTTTGAAATTTATCTGCACCA
CTGGTAAACTGCCTGTGCCTTGGCCGACGTTGGTTACGACCCTGACCTATGGAGTCCAATGTTTCTCGCGGTATCCG
GATCACATGAAACAGCATGACTTCTTCAAGAGCGCAATGCCCGAGGGTTATGTGCAAGAGCGCACTATCTCGTTTAA
GGACGATGGCACTTATAAAACTCGCGCAGAAGTCAAGTTCGAGGGTGATACTTTAGTCAATCGTATAGAACTTAAAGG
TATAGACTTCAAGGAAGATGGAAACATCCTGGGCCATAAATTAGAATATAATTTTAATAGCCACAATGTATACATTACCGC
AGACAAACAAAAAAACGGTATAAAAGCTAACTTCAAAATTCGTCATAACGTGGAAGACGGATCGGTACAACTCGCCGA
CCATTACCAGCAAAACACTCCAATAGGTGATGGTCCGGTGCTGCTGCCGGATAACCACTATCTTTCTACTCAAAGTGT
CCTAAGTAAAGACCCGAACGAAAAAAGAGATCATATGGTATTATTAGAATTTGTGACAGCGGCAGGGATTGGATCCGA
TGACGACGATAAGGAATTCGGACGCGGTATTCGATATACGATGTACAATCTTGGCGATATCTTCGTTGACAAGCTGGA
ATCCATGTGGAACTCGACTAAAATCAGCACAAAAGGTATTCGCCTGACCCATAATATCCGCAAATTACGGTCAATTAAA
GAGGAGCAGGAGCGCAAGCTCAGTGGGCGTGTCCTAGAGTTACGTGAATCGTATCCAGAATTGGAAATCTTTAAAGA
CGACGAACTGTCTAAACTGTTTAGTGAAATTGATGCGATAAACCGCGAACTGGATAGCTATATCGAAGAACGTGATGA
AATTCTGTACCCGACCGGTCGTTCTGTGGCTAGCCATCACCATCATCACCACTAACGGCCG

Recombinant Protein Expression

The sfGFP-Mad10trunc-His fusion protein was expressed recombinantly in the cytoplasm of *E. coli* strain BL21(DE3) using standard protocols. The expression culture (LB medium; tryptone 10 g L^{-1}, yeast extract 5 g L^{-1}, NaCl 10 g L^{-1}) was inoculated from starter cultures, with an OD_{600} of 0.1, and incubated at 37 °C and 210 rpm. Protein expression was induced by the addition of isopropyl-β-D-thiogalactopyranoside (1 mM final concentration) when the OD_{600} reached a value between 0.6 to 0.8. Following induction, the culture was grown at 37 °C and 210 rpm for 4 h, after which the culture was harvested by centrifugation (4,000 g, 15 min, 4 °C). To purify the protein, the cell pellet was resuspended in loading buffer (50 mM NaH_2PO_4,/NaH_2PO_4, pH 8, 300 mM NaCl, 15 mM imidazole). To lyse the cells, the suspension was passed through a high-

pressure homogenizer (Emulsiflex-B15; Avestin) and then cleared by centrifugation (20,000 g, 60 min, 4 °C). The cleared lysate was passed through a 0.22 µm filter and loaded onto Ni^{2+}-NTA columns (His GraviTrap™; Cytiva) pre-equilibrated with loading buffer. The columns were then washed with 10 column volumes (CV) of loading buffer and 20 CV of washing buffer (50 mM NaH_2PO_4,/NaH_2PO_4, pH 8, 300 mM NaCl, 30 mM imidazole). The protein was eluted with 6 CV of elution buffer (50 mM NaH_2PO_4,/NaH_2PO_4, pH 8, 300 mM NaCl, 200 mM imidazole). The protein was dialyzed against interaction buffer (50 mM NaH_2PO_4/Na_2HPO_4, pH 8, 50 mM NaCl) (12-14 kDa MWCO, Spectra/Por®; Spectrum), aliquoted, and stored at -80 °C until further usage.

SDS-PAGE

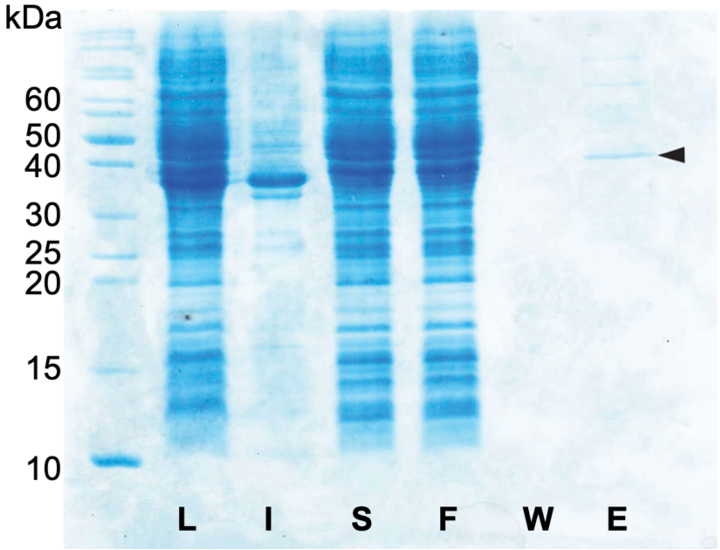

Figure S2: Reducing SDS-PAGE (15%) showing expression and purification of sfGFP-Mad10trunc-His with L: lysate, I: insoluble protein, S: soluble protein, F: flow-through, W: washing buffer, and E: eluted protein. The black arrow indicates the sfGFP-Mad10trunc-His band on the gel.

References

[1] A. Pohl, S. A. E. Young, T. C. Schmitz, D. Farhadi, R. Zarivach, D. Faivre, K. G. Blank, "Magnetite-binding proteins from the magnetotactic bacterium Desulfamplus magnetovallimortis BW-1" *Nanoscale* **2021**, *13*, 20396–20400.

[2] X. Li, G. Zhang, N. Ngo, X. Zhao, S. R. Kain, C. C. Huang, "Deletions of the Aequorea victoria green fluorescent protein define the minimal domain required for fluorescence" *J Biol Chem* **1997**, *272*, 28545–28549.

[3] H. K. Kim, B. K. Kaang, "Truncated green fluorescent protein mutants and their expression in Aplysia neurons" *Brain Res Bull* **1998**, *47*, 35–41.
